# Simulation of emitter discharge along drip laterals under drip fertigation system using artificial neural network

**DOI:** 10.1371/journal.pone.0326948

**Published:** 2025-07-08

**Authors:** Oluwaseun Temitope Faloye, Smart Idumoro Samuel, Abiodun Afolabi Okunola, Viroon Kamchoom, Natdanai Sinsamutpadung, Oluwafemi Adeyeri

**Affiliations:** 1 Department of Water Resources Management and Agrometeorology, Federal University, Oye-Ekiti, Ekiti, Nigeria; 2 Department of Civil Engineering, Landmark University, Omu Aran, Kwara, Nigeria; 3 Department of Agricultural and Biosystems Engineering, Landmark University, Omu Aran, Kwara, Nigeria; 4 SDG Food Security Research Group, Landmark University, Nigeria; 5 Excellent Centre for Green and Sustainable Infrastructure, School of Engineering, King Mongkut’s Institute of Technology Ladkrabang, Bangkok, Thailand; 6 ARC Centre of Excellence for the Weather of the 21st Century, Fenner School of Environment and Society, The Australian National University, Canberra, Australian Capital Territory, Australia; Indian Council of Agricultural Research - Indian Agricultural Research Institute, INDIA

## Abstract

Simulation of emitter discharge under a drip fertigation system is important for capturing the variation in water and nutrient distribution to crops. This is important for an effective design and irrigation management for agricultural crops. Moreover, the field discharge measurements are laborious and time-consuming, hence the need for the development of a representative model. The application of artificial neural network to simulate drip emitter along drip laterals is new in the field of flow measurement under drip irrigation. The purpose of this study is to predict the emitter discharge along drip laterals using artificial neural network (ANN) and evaluate the performance of the model. The input parameters fed into the ANN include; pipe length away from the fertigation source, elevation heads and distance of emitter point along the laterals. The field measured discharge was considered as the output. Evaluation parameters considered for the designed drip fertigation system indicated high efficiency, in the range between 81 and 98%. Interaction effects were observed between the pipe length and elevation head on the uniformity coefficient (CU) and emitter discharge. When all data were simulated, the ANN model simulated the emitter discharge accurately and precisely along the drip laterals, with R^2^ value ranging between 0.81 and 0.89, while the normalized root mean square error (NRMSE) was mostly below 20%, thus indicating a good prediction. The mean absolute error ranged between 0.034 and 0.048. Therefore, the ANN model was efficient for capturing the variation in emitter discharge well under the drip fertigation system.

## 1. Introduction

Irrigation is the artificial application of water to soil for crops’ use [1–5]. A network of facilities and machinery called an irrigation system has been made to efficiently and effectively distribute water to landscapes and crops [6]. The ability of the irrigation system to save more water depends on the type of irrigation system adopted. According to the study by Patel and Rajput [7], the classes of irrigation systems include sprinkler, drip, centre pivot, subsurface, flood and furrow irrigation. Among these above-stated methods, drip irrigation has been recognized as the most effective approach of enhancing or improving the crop water use and nutrient use efficiency. The system enhances crop yields and quality, optimize water use, reducing labor and energy costs, improving soil health and fertility, and supporting maintenance of landscape and aesthetics [7]. These benefits are achieved since it allows moderate flow rate directly to the root zone of crops thereby, meeting the crop water requirement [7].]

The system uses a network of hydraulic components like pipes, tubes and emitters to deliver water straight to the root zone of the plant [7–12]. These hydraulic components must be well designed to derive the best benefit from the drip irrigation system. Several researchers [10,13,14] have been carried out to evaluate the hydraulic performance of a designed irrigation system. These above-stated studies considered the variations in the discharges along the emitters. This variations in the discharges were reported to have effect on water and fertilizer distribution of the system and consequently affecting the growth and yield of crops [15]. Therefore, a well-designed drip fertigation system will offer a precise, efficient, and sustainable way to manage water and nutrient applications.

Considering the importance of effective management of irrigation water and fertilizer on crop growth and yield, there is a need to evaluate the hydraulic performance of any designed drip irrigation system [10,13,16]. These above-stated researchers acknowledged hydraulic variables like flow rate (Q), coefficient of variation (CV), coefficient of uniformity (CU), emission uniformity (EU), emission flow variation (EFV), and water application efficiency (AE) as parameters required for evaluating the hydraulic performance of a drip irrigation/fertigation system. The above-stated hydraulic variables have been scarcely applied under a drip fertigation system.

Evaluating the performance of a designed drip fertigation system is important, to determine and know the variation in nutrient distribution under a drip irrigation system. This is because the dilution effect of two different substances (fertilizer nutrient and water) might also cause some changes in the rheological and hydraulic behaviour of any fluid [17–26]. In addition, other factors which may affect/alter the hydraulic performance of a drip fertigation/irrigation system include the pipe and drip lateral length, and the elevation head.

Recent studies [27–31] have acknowledged that these above-mentioned parameters have effects on the discharge from drip emitter. Moreover, the measurement of the emitter discharge along the drip laterals are required for the determination of the performance evaluation. However, the process of obtaining these discharges are always laborious, costly (cost of the irrigation gadgets) and time-consuming [17,32–37], hence necessitating the development of a model that can represent the field measurement. Moreover, the development of a model that can capture the variability in the discharge measurement along the drip lines will help in proper crop budgeting, since it is already well known that water distribution of any system is dependent on emitter discharge variability, and consequently on the crop yield [15].

Till date, there is dearth of studies on the development of model for the prediction of emitter discharge along laterals using AI approach, particularly with the use of ANN. Previous studies on the prediction of emitter discharge along emitter discharge has primarily been with the use of analytical approach [38–41]. These above-stated studies reported high accuracy in predicting the emitter discharge. However, the analytical method had been acknowledged to be complex in analyzing and characterizing hydraulic systems [42,43]. Another approach which is commonly used in hydrological modelling is the regression model. Despite its simplicity, it is mostly condition specific, which may hinder its applicability under varying hydraulic conditions like wide range of input parameters, drip irrigation system type, emitter discharge and pipe length [44]. Therefore, there is a need to develop a model like ANN, which has a wide application, irrespective of the above stated scenario. In this study, the artificial neural network (ANN) was tested for the prediction of the emitter discharge. Previous studies that tested ANN for hydrological modelling under some irrigation methods, only considered it for wetting fronts prediction [45–47]. These previous studies did not consider the prediction of the emitter discharge, which is also an important factor affecting wetting front dimensions [45–47]. The ANN was selected due to its wide applicability in various discipline and systems [48–54]. Moreover, it has been successfully used for prediction in situations that involve dynamic system, hydrology and agriculture [55–61], which is similar to the condition in this study, since the discharge from the emitter is dynamic, that is, it changes with time. Therefore, the objectives of the study are to (i) evaluate the hydraulic performance of a drip fertigation system, as influenced by pipe length and pressure head (elevation height); and (ii) evaluate the performance of ANN model in accurately and precisely predicting emitter discharge along drip laterals under different hydraulic scenarios – pressure head (elevation height) and pipe length.

## 2. Materials and methods

### 2.1 Description of the study area

**Fig 1:**
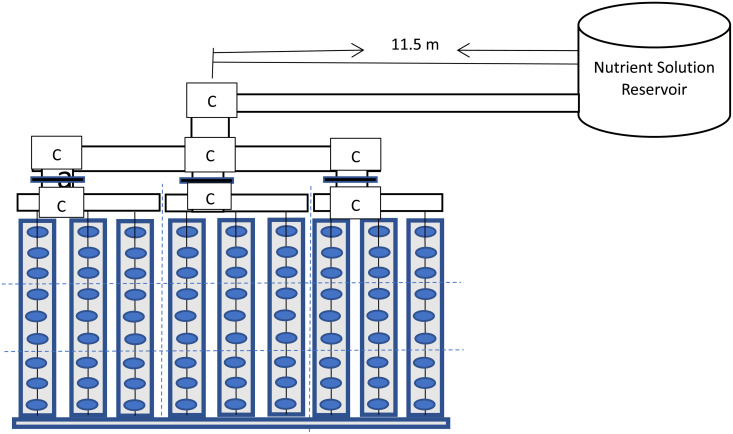
Schematic diagram of the drip fertigation system.

The study was carried out at the teaching and research farm, Landmark University, Omu Aran. The town, Omu-Aran, is a town in the sub-humid agro-ecological zone of Southern Guinea Savanna, Kwara State, Nigeria. It is situated at latitudes 8° 8′00′′N and longitude 5° 6′00′′ E and at an elevation of 564 meters above mean sea level. The climate of Omu Aran is primarily tropical maritime, with a lengthy rainy season, mild weather all year long and an average daily temperature between 16 and 32° C. According to Opeke [30], Raphael et al. [55] and Kayode et al. [62], Omu Aran receives the most rainfall across the state, since the town is the nearest among other towns in Kwara State to the rainforest and derived savanna in the middle belt of Nigeria. Depending on the difference in hot and cold weather during seasonal changes, the average rainfall depth is between 600 and 1200 mm, and it is distributed across 6–8 months of the year. Fig 1 shows the location of Omu Aran in Kwara State, Nigeria. The area has a very deep-water table depth, as such upward movement of water by capillarity is prevented. The soil textural type of the study location is sandy soil. The bulk density range between 1.35 and 1.48 g cm^-3^, while the soil porosity ranged between 45 and 49.1% [3].

### 2.2 Experimental setup and procedure

**Fig 2:**
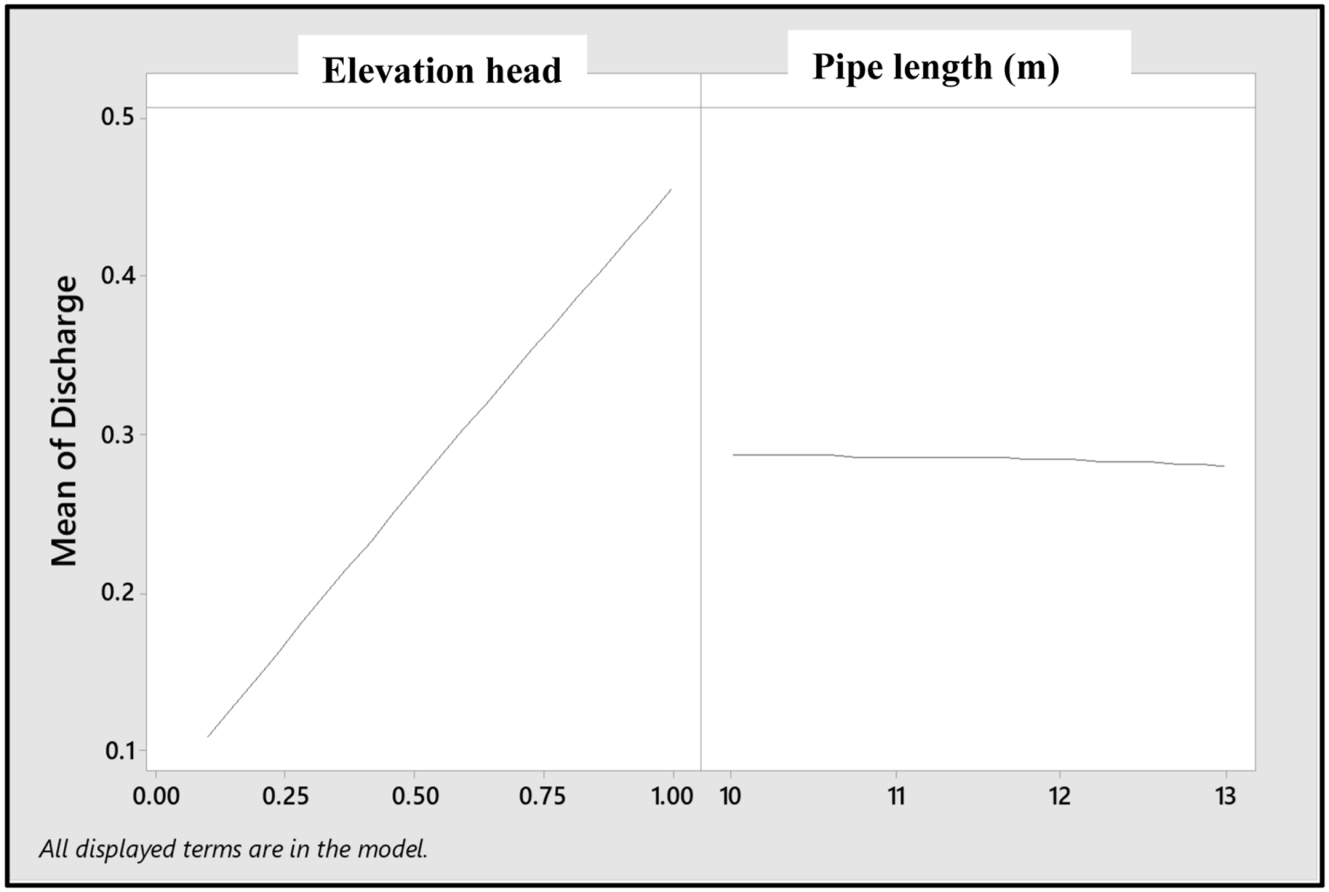
Main effects of elevation head and pipe length on the emitter discharge.

The experimental setup of the drip fertigation system was done in a screen house in Landmark University, Omu Aran. It comprises of a tank/ reservoir that contained the nutrient solution (a mixture of liquid fertilizer and water in the ratio 1 is to 1000). The liquid fertilizer used in the drip-fertigation was super-gro with the mixture in the ratio of 1 litre of water to 1 mL of liquid fertilizer obtained within the study area, Omu-Aran. The drip irrigation laterals are spaced at 30 cm and was a point source type. During the drip-fertigation system installation, one inch (1“) PVC pipe was used as the main line and the lateral lines from the drip-fertigation system was connected to the sub-main pipe of size ¾ inch. The reservoir (over-head tank) was positioned at distances of 10, 11.5 and 13 m away from the valve controlling in the laterals (Fig 2). At each considered distance away from the reservoir, there was three drip lines (rows), each line serving as a replicate, thus producing a total of 3 replicates in each distance (pipe length) away from the reservoir (source). In each lateral line, there was 9 emitters, while 30 cm intra- and inter-spacing were maintained. The average value of the discharge measurement from the 9 emitters was found to represent measurement from a replicate using the gravity fed fertigation system.

During the trial test, catch cans were positioned below the drip emitters and the amount of water harvested by the cans were measured at 1 hour of discharge. This trial test was repeated at different elevation heights of 0.1, 0.5 and 1m. Therefore, the experimental design adopted with this type of procedure was a factorial design, comprising of 3 elevation head, 3 pipe length, 9 distance of emitter points along the drip laterals and 3 replicates, therefore resulting in 243 measurements. However, the average discharge from the 3 replicates were obtained, and further used for the modelling, thus a total of 81 discharge data were input into the model (3 elevation head x 3 pipe lengths x 9 distance of emitter points along the drip laterals. During the trial test, at exactly 1 h, the control valve is closed, and the discharges collected in the catch cans were measured using a well calibrated measuring cylinder, and the discharges were expressed in litre per hour (L h^-1^). The drip lateral type used in this study belongs to the Jain brand, spaced at 30 cm interval with flow rates ranging between 0.26–1.36 L h^-1^, based on the manufacturer specification.

### 2.3 Evaluation of hydraulic performance of the drip irrigation system

**Note:** R represent replicate

According to the studies by Sharu [10], and Yildirim and Agiralioglu [13], the flow rate discharge (Q), emission uniformity (EU), coefficient of uniformity (CU) and application efficiency (AE) are important to be taken into account when determining the hydraulic performances of a drip irrigation system. These parameters were estimated using equations 1–4.

#### 2.3.1 Emitter flow discharge.

Emitter flow discharge (Q) is a critical parameter in a drip system’s design and operation, as it directly affects water application uniformity, crop water requirements, system pressure, energy consumption and water distribution efficiency [16,60]. It is determined by volumetric measurement, that is, the amount of water released by the system over a predetermined period of time. In this study, the volume of the emitters’ discharges was measured using a measuring cylinder and divided by the time of system operation, expressed in litre per hour (L h^-1^) as shown in Equation 1 [62].


$$Q=\frac{V_{w}}{t}$$ (1)

Where the discharge of each emitter, Q is given in L h^-1^, V_w_ is volume of liquid collected from each emitter (l) and t is specific time of operation (h).

#### 2.3.2 Coefficient of uniformity.

The coefficient of uniformity (CU) is a key performance indicator for a drip irrigation system [63,64]. It measures the uniformity of water distribution throughout a field or crops. According to studies by Sharu and Razak [61], a higher CU of 90% or more, indicates a more uniform water application, guaranteeing accurate and effective water delivery to the crops, while a lower CU indicates greater variability. In this study, CU was evaluated base on the variation in water application [25], using the equation 2, prescribed by ASAE [65].


$$\text{CU}=100(1-\sum \frac{\Delta q}{qn})$$ (2)

Where Cu is the uniformity coefficient (%), ∆q is the average deviation of individual emitters discharge (L h^-1^), q is Average discharge (L h^-1^) and n is the number of observations.

#### 2.3.3 Emission uniformity.

Emission uniformity (EU) measures the consistency of water emission along a drip irrigation lateral (tube). It shows the uniformity of the distribution of water among the emitters. According to Elamin et al. [28], by evaluating EU of a drip system, farmers and/or irrigators can optimize the design of the system, the operation and maintenance to ensure uniform water application and maximize crop yields. Also, that a higher EU indicates more uniform water emission, while a lower EU indicates greater variability in water application. EU is expressed as a percentage of the minimum emitter discharge to the average emitter discharge, using equation 3, defined by ASAE [65].]


$$EU=100\left(\;\frac{q^{n}}{q^{a}}\;\right)$$ (3)

Where EU is the emission uniformity (%), q^n^ is the average discharge of the lowest one fourth of all discharges (L h^-1^) and q^a^ is the average emitter discharge of all the (L h^-1^).

#### 2.3.4 Application efficiency.

The percentage of water applied for real plant usage in a drip irrigation system is known as application efficiency [66]. That is, the ratio of water needed at the root zone to the total amount applied. It displays the effectiveness of irrigation water application, i.e., the proportion of water applied that is retained in the root zone and made accessible for plant use [67]. The application efficiency of the drip fertigation system was determined using equation 6 [68].


$$AE=\frac{q_{\min}}{q_{avg}}$$ (4)

Where AE is the application efficiency (%), q_min_ is the minimum emitter discharge (L h^-1^), q_avg_ is the average emitter discharge (L h^-1^).

### 2.4 ANN modelling approach used for the prediction of the emitter discharge along the drip laterals

#### 2.4.1 Model data input.

The pipe length (10, 11.5 and 13 m), distance of emitter along the drip laterals (0.3, 0.6, 0.9, 1.2, 1.5, 1.8, 2.1, 2.4 and 2.7 m) and elevation head (0.1, 0.5 and 1 m) were used as the input data for the emitter discharge prediction. These range of pipe length and elevation heads have been similarly applied in previous studies [28,69]. These combinations of arrangement form 3 x 9 x 3, and total 81. These 81 data were pooled together, and used as data input, that is, 81 data entries (3 pipe lengths x 9 different distance of emitter along the drip laterals x 3 elevation heads), and were used for the prediction of the emitter discharge along the emitter discharge. The data scale of 81 used in this study has been successfully used for the estimation of wind drift and evaporation loss under sprinkler irrigation [70]. To obtain average values for the 9 different points of emitter along the drip laterals, the average value of the discharge from 3 replicates at each distance from the fertigation source was considered (Fig 2). For each pipe length, there are three replates for each emitter discharge, and the average value was recorded. In a similar hydrology study, Faloye et al. [59]] applied 56 set of data to successfully model moisture movement in soil while Sharu and Ab Razak [61] used a total 20 data. The higher the set of data used the more reliable the developed model, which gave the present study an advantage, since larger data-set were employed.

The overall consideration was to test the capability and robustness of the ANN in capturing the variability in the emitter discharge along the drip emitters. In addition, after the overall prediction, the prediction of the emitter discharge at each elevation head was carried out, and producing 27 outcomes (3 pipe lengths x 9 drip laterals). The fraction of the data that was used for training, validation and testing were 70, 15 and 15%, respectively. The descriptive statistics of the data input and the field measured emitter discharge is as illustrated in Table 1.

**Table 1:** Summary statistics of input data used for the emitter discharge prediction.

	Elevation height (m)	Pipe length (m)	Emitter point along emitter	Discharge (L/h)
MAX	1	13	2.7	0.60
MIN	0.1	10	0.3	0.088
MEDIAN	0.5	11.5	1.5	0.263
MEAN	0.53	11.5	1.5	0.275
STD	0.37	1.23	0.78	0.04
CV				0.15

#### 2.4.2 Theory of ANN and the procedure followed in the prediction using ANN.

ANN is a tool that mimics the function of biological neurons using computational means. The ANN model’s architecture dictates how well it works. The number of input nodes, output nodes, and hidden layer are all part of the ANN design. The input-output relationship is often obtained by training the model throughout the construction of an ANN model, which is crucial to determine the ideal weights.

Calculating the output variables from the input variables, comparing the measured output with the output of the ANN model, and modifying the weights and bias for each node are all steps involved in the training process. The goal of the adjustment is to minimize the discrepancy between the calculated and measured output values. The model is then verified and put to the test. The three crucial phases of model development are training, validation, and testing, with the shuffle-split approach applied. MATLAB version 2013 was used to develop an artificial neural network model for the emitter discharge along the drip laterals. The ANN model was trained using the Feed-Forward Back Propagation (FFBP) algorithm in conjunction with the Levenberg-Marquardt training function. Due to its widespread use in agriculture, hydrological and hydraulic modeling research [69–72], the feed forward neural network back propagation training algorithm was used to create the ANN model. This study employed the Tan sigmoid Transfer Function (TANSIG), Log sigmoid Transfer Function (LOGSIG), and the PURLIN transfer function. The essence of testing these three transfer functions was to select the one that gave the best results, in terms of accuracy and precision. The hidden layer was tested between 1 and 15 neurons, since there is no rule for using a particular number of neurons for the hidden layer. The hidden layer with neuron of 10 gave the best result, in terms of lowest RMSE and MAE.

The LEARNGDM learning function—which stands for gradient descent with momentum weight and bias learning function—was used to model this ANN. Learning is the process by which a neural network uses the right parameter modifications to adapt to a stimulus and produce the intended response. This method of learning is comparable to the methods employed by other soil science, hydraulics and hydrological researchers [62–64].

### 2.5 Statistical analysis

The mean values of various parameters for the hydraulic performance of the drip fertigation system at different elevation heads were subjected to a One-way ANOVA (analysis of variance) to statistically determine their significant differences using Mini-Tab, version 17. The significant differences in their mean values were compared using Tukey method at 95% confidence level. Two-way ANOVA was also applied to determine the main and interactive effects between the pipe length from the reservoir and elevation head on the emitter discharge. In addition, the developed model evaluation was carried out for the model precision assessment using the coefficient of determination (R^2^), which is a common indicator commonly used for model evaluation in sciences [62–80]. For the accuracy terms of the ANN model, the root means square error (RMSE), mean absolute error (MAE) and the normalized root mean square error (NRMSE) was determined to evaluate the performance of the ANN model in predicting the emitter discharge along the emitters [81–88] (equations 5–7).


$$MAE=\frac{1}{n}\sum_{i=1}^{n}|M_{i}-S_{i}|$$ (5)

$$RMSE=\sqrt{1/n\sum (M_{i}-S_{i}{)}^{2}}$$ (6)

$$NRMSE=\frac{{\sqrt{1/n\sum (M_{i}-S_{i})}}^{2}}{\overset{―}{M}}$$ (7)

Where M_i_ and S_i_ are measured and predicted data, respectively. $\overset{―}{M}$ is the mean value of M_i_, and n is the number of observations.

The closer the RMSE and MAE tend to zero, the more accurate the model, and the more NRMSE and R^2^ approaches 1, the more accurate and precise the model. NRMSE of < 10% is excellent, 10–20% is good, 20–30% is fair and greater than 30% is poor.

## 3. Results and discussion

### 3.1 Emitter discharge of the drip fertigation system

The emitter flow discharge (Q) through gravity fed drip-fertigation system in this study increases with increasing elevation head (H) (Table 2). The highest discharge, 0.450, 0.456 and 0.457 L h^-1^ were recorded for H at 1m at pipe lengths of 10, 11.5 and 13.5m, respectively. For H at 0.1 and 0.5 m, the discharges ranged between 0.105 and 0.27 L h^-1^ (Table 2). The analysis of variance (ANOVA) revealed that there was no significance in the emitter discharge with pipe length (P > 0.05), while the elevation head significantly (P < 0.05) affected the discharge from the emitter. The main effect of the pipe length was insignificant (P > 0.05) on the emitter discharge, while the main effect was strongly significant (P < 0.0001). Moreover, the main effect plot, which reveal the overall trend of the pipe length and elevation head effects on the emitter discharge showed that as the distance advances (pipe length) the drip emitter discharge reduces (Fig 2), with the lowest emitter discharge observed at pipe length 13 m.

**Table 2:** Emitter flow discharge (L h^-1^) of the drip fertigation system.

Pipe length (m)		Elevation head (m)	
	**0.1**	**0.5**	**1**
**10**	0.110^c^	0.270^b^	0.450^a^
**11.5**	0.107^c^	0.267^b^	0.456^a^
**13**	0.105^c^	0.253^b^	0.447^a^
**Main Effects**			
Pipe length	ns	ns	ns
Elevation head	****	****	****
**Interaction Effect**			
Pipe length * Elevation head	ns	ns	ns

**Note:** The means with the same letter(s) in the same row do not differ significantly (p ≥ 0.05)

The overall slight reduction in the discharges as the pipe length advances could be attributed to minor losses that occur in valves, pipe fittings and bends [89,90]. It’s well established from the Darcy-Weisbac equation (equation 8) that as the length of pipe increases the friction head loss increases. Moreover, as the friction head loss increases, there is reduction in the emitter discharge [91].


$$H_{f}=\;\frac{fLV^{2}}{2gD}$$ (8)

The minor losses may become more and intense as the pipe length increases, hence resulting to reduced discharge. This is because more minor losses along the pipe length could affect water transmission and distribution due to loss pressure energy, and consequently alter the emitter discharge [91]. Another possible explanation could be attributed to frictional loss, which may occur between the water flowing through the pipe and the pipe wall [92]. The more the length of the pipe, the more the possibility of the frictional loss to affect the emitter discharge. Fig 2 also showed that the emitter discharge markedly increases as the elevation head increased. This approach revealed that instead of using pump to aid the flow, the elevation head could be simply increased to enhance the flow (discharge). The interaction effects between the pipe length and elevation head were insignificant (P > 0.05) (Table 2). This implies that the elevation head does not significantly (P > 0.05) depend on the pipe length to influence the emitter discharge base on the pipe length of 10–13 m considered in this study. For further study, longer distance could be considered.

**Fig 3:**
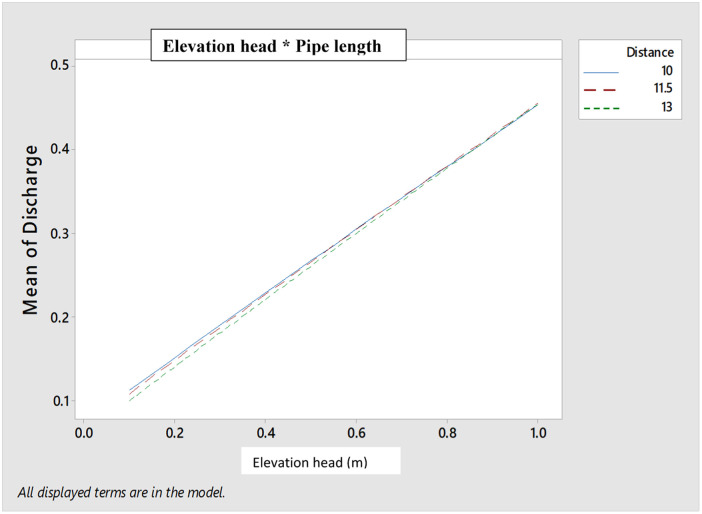
Interactive effects between elevation head and pipe length on the emitter discharge.

Despite the insignificant interaction effects between the elevation head and pipe length, there still exist interactions between these two factors (Fig 3), but their interactions were not only significant (P > 0.05). This again revealed longer length of pipe could be tested to show if at longer length, significant (P < 0.05) interaction effects could occur.

### 3.2 Performance evaluation of the designed drip fertigation system

The standard ranking of installed drip irrigation performance evaluation is given in Table 3. The values obtained in this study has been compared to the American Society of Agricultural Engineers [65].

**Table 3:** Range of values and classification of hydraulic performance of a drip irrigation system.

Parameter	Values	Performance indicators
Coefficient of uniformity (CU)	≥ 90%	Excellent
	80 - 90%	Good
	70 - 80%	Fair
	60 - 70%	Poor
	< 60%	Unacceptable
Emission uniformity (EU)	≥ 90%	Excellent
	80 - 90%	Good
	70 - 80%	Fair
	≤ 70%	Poor
Application efficiency (E_a_)	≥ 90%	Excellent
	80-89%	Good
	70-79%	Fair
	< 70%	Poor

Source: American Society of Agricultural Engineering [65]

For the coefficient of uniformity, in this study, H at 0.1 m gave the highest uniformity coefficient (CU) of 98.6% at 13 m pipe length, followed by 98.5 and 97.9 at 11.5 and 10 m pipe length, respectively. The CU estimated in this study for the three elevation heads at the different pipe lengths gave excellent classifications (Table 4) as prescribed by ASAE [65] in Table 3 above. The study of AL’Amound [93] reported that a higher CU, which is greater or equal to 90% (≥ 90%) indicated a more uniformed water application, while a lower CU, that is less than 70% (< 70%) indicates greater variability or poor water distribution uniformity, which may eventually lead to increased wastage of water. Similar trend with respect to the pipe length were observed for H at 0.5 and 1 m, respectively. According to a study carried out by Christiansen [94], CU value ranges from 0 (least value) to 100% (highest value) and that CU of 90% or higher value is generally considered acceptable for a well-designed system. The values of CU obtained in this study, which are within the range of 97.3 and 98.6%, are higher than the range between 86.3 and 94.6% reported by Elamin et al. [28] using pressure head between 0.4 and 1 m. The range of CU reported in our study also compare similarly well with the range of 98.23 and 98.52% reported by Sharu [10].

**Table 4:** Effects of the pipe length and elevation heads on the performance evaluation parameters.

		Elevation head (m)	
**Pipe length (m)**	**0.1**	**0.5**	**1**
	**Emission Uniformity (EU)**		
**10**	81.9a	81.2a	82.3a
**11.5**	87.5a	81.2ab	80.5b
**13**	91.5a	80.0a	82.6a
	**Coefficient of Uniformity**		
10	97.9a	97.5a	97.5a
11.5	98.5a	97.3b	97.8b
**13**	98.6a	97.3a	97.9a
	**Application Efficiency**		
10	80.9a	81.7a	82.3a
11.5	88.6a	80.6a	81.6a
13	88.4a	81.1a	81.9a

**Note:** The means with the same letter(s) in the same row do not differ significantly (p ≥ 0.05).

The application efficiency (AE) evaluated in this study ranged between 80.6 and 88.6 for elevation heads of 0.1, 0.5 and 1 m, respectively (Table 4), which is considered good. The ANOVA result revealed that there was no significant difference (P > 0.05) in the effects of the elevation heads on the AE. The study reported by Howell [95] described application efficiency of a well-designed system to be excellent with values greater than or equal to 90% (≥ 90%). Similarly, Mirjat et al. [66] reported that AE with value greater than or equivalent to 90% indicates a more efficient drip irrigation system. These above-mentioned researchers posited that AE value less than 70% (< 70%) is considered low, indicating reduced application efficiency, large water waste and may consequently result to reduced crop yield.

The emission uniformity (EU) obtained in this study ranged between good and excellent, with higher performance mostly noted at the 0.1 m elevation head. The study carried out by AL’Amound [93] posited that higher emission uniformity (EU), which is greater or equal to 90% (≥ 90%) indicated more uniform water emission, while a lower EU, that is less than 70% (< 70%) indicated greater variability in water application. In this study, the 0.5 and 1 m elevation heads produced EU that is good for the various irrigation distances (pipe lengths), while highest values were observed at 0.1 m elevation head. These classifications were based on the recommendations prescribed by ASAE [65] (Table 3). The EU values reported in this study compare favourably well (and even slightly higher) with the value of 80.21% reported by Patle [37] at the 1 m elevation head, and a range of 81.0–92.6% reported by Elamin et al. [28] using pressure head between 0.4 and 1 m. The results as shown in Table 4, indicates that emission uniformity for gravity flow drip fertigation system increases with increasing elevation head. This observation is in agreement with the submission of Elamin et al. [28] and El-Mansy et al. [38]. It can be deduced from this study that lower elevation head produced higher emission uniformity and coefficient of uniformity. Similar observations were observed by some other researchers [38,61]. Similar to the CU and EU, higher application efficiency was mostly observed at the lower elevation head. The improvement in the CU and EU at the lower elevation head could be attributed to the gentle discharge of the emitter, which could lead to the minimization of water wastage improves application efficiency.

In order to ascertain the overall effects of the pipe length and elevation heads on the performance of drip fertigation system, the main and interactive effects of the pipe length and elevation heads on the coefficient of uniformity have been considered.

### 3.3 Main and interactive effects of the pipe length and elevation heads on the coefficient of uniformity (CU)

**Fig 4:**
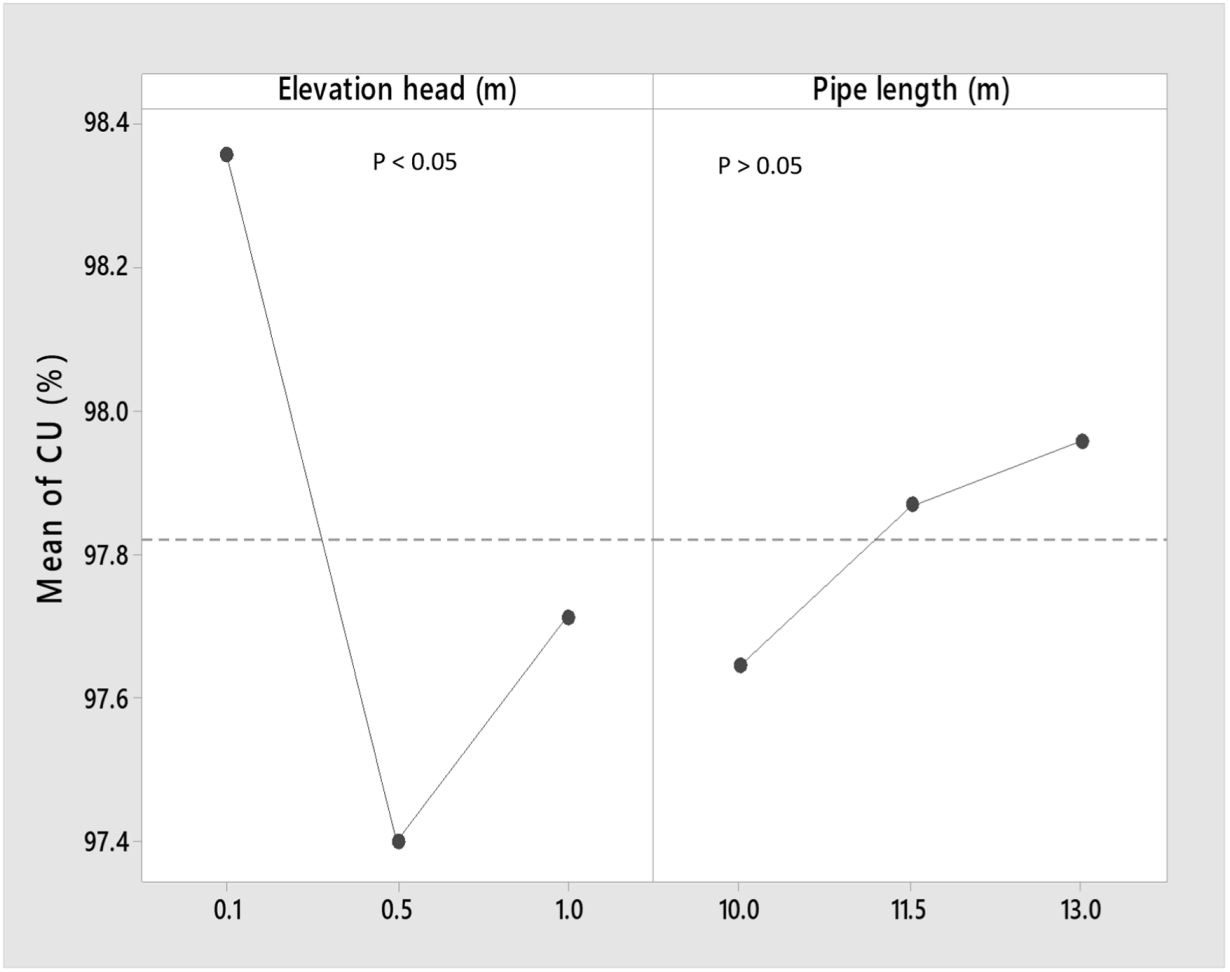
Main effect of the elevation head and pipe length on the CU.

**Fig 5:**
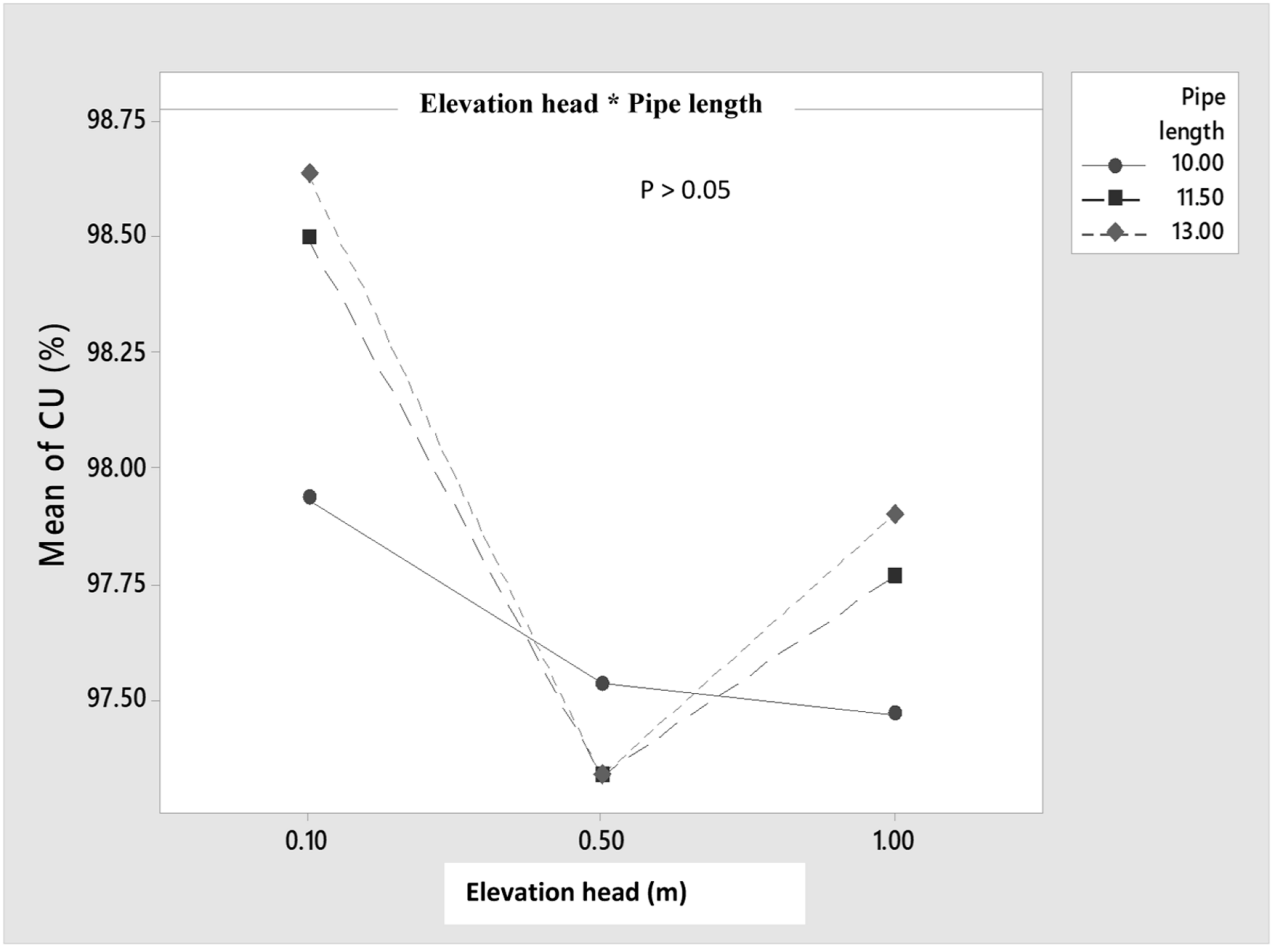
Interactive effects of the elevation head and pipe length on the CU.

The main and interactive effects between the pipe length and the elevation head is revealed in Figs 4 and 5. For the main effect, the Fig 4 revealed that the highest CU was obtained at the lowest elevation head, while lower CU was obtained at higher heights of 0.5 and 1 m. The main effect of the impact of elevation head on the CU was significant (P < 0.05) on the CU, while a negative trend was observed, revealing that the CU increases with decreasing elevation head. This may imply that the gentle flow of water could contribute to efficient water distribution, which may enhance the water use efficiency of a possible horticultural crop cultivated under fertigation condition. This is important since there is every effort to increase the profitability of farmers [96,97], which could be achieved by determining optimal conditions of any system [98–102].

In addition, the main effect of the pipe length was insignificant (P > 0.05) on the CU, with the CU increasing with the increasing pipe length. The minor losses which might have intensified with increasing pipe length resulted to reduced discharge [92]. This gentle flow due to some minor losses at the longer pipe length or reduced energy in the water may have resulted to enhanced CU at 11.5, 13 m pipe length, as compared to the 10 m pipe length.

The interaction effects between the elevation head and the pipe length are indicated in Fig 5, which showed that interaction occurred between the pipe length and the elevation head. But based on the ANOVA Posthoc test, the interactive effects were insignificant (P > 0.05). This insignificant difference (P > 0.05) effect observed could be attributed to the length of pipe used for the study, as longer pipe length could result in much significant impact in their interaction with the elevation head.

Overall, the comprehensive analysis of the interrelationships between the pipe length, elevation head and the CU is important since the CU is also well linked and connected with the emission uniformity and application efficiency, meaning that better uniformity will lead to higher application efficiency [96]. Moreover, some researchers [95,103] already posited that comprehensive evaluation of any irrigation system is important for the optimal productivity of any cultivated crop under irrigation and fertigation conditions for enhanced agricultural productivity.

### 3.4 Simulation of the emitter discharge along the drip laterals

**Fig 6:**
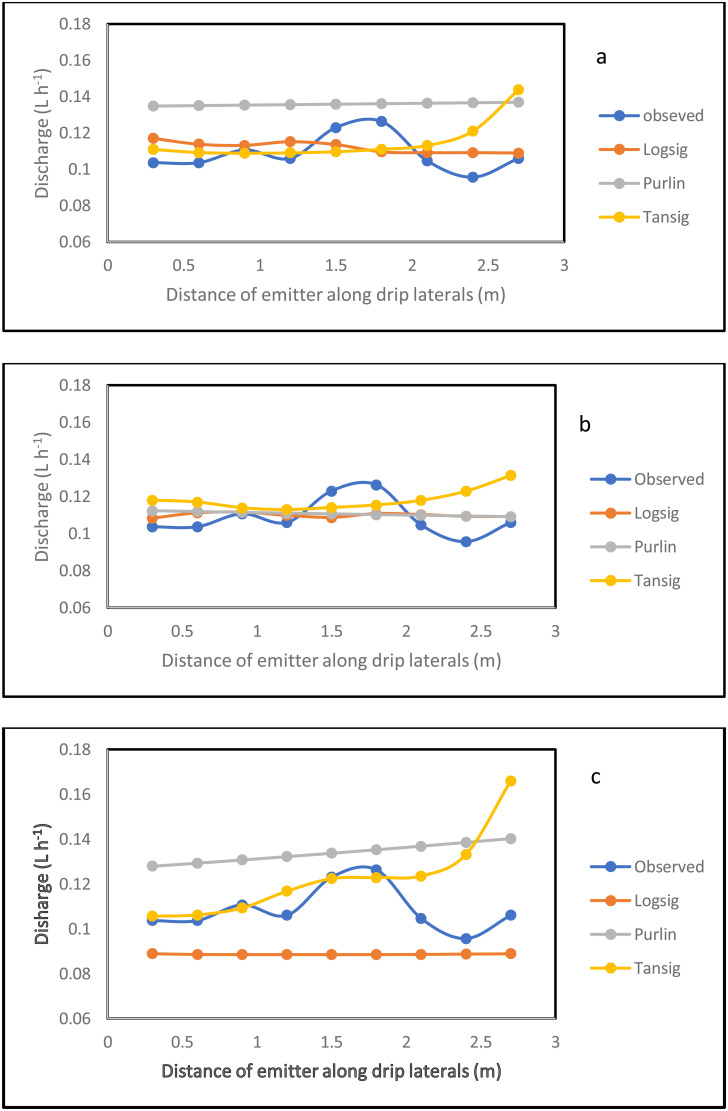
Simulation result for the emitter discharge along the drip laterals at 0.1 m elevation height and pipe length of 10 m (a), 11 m (b) and 13.5 m (c) pipe length using ANN.

**Fig 7:**
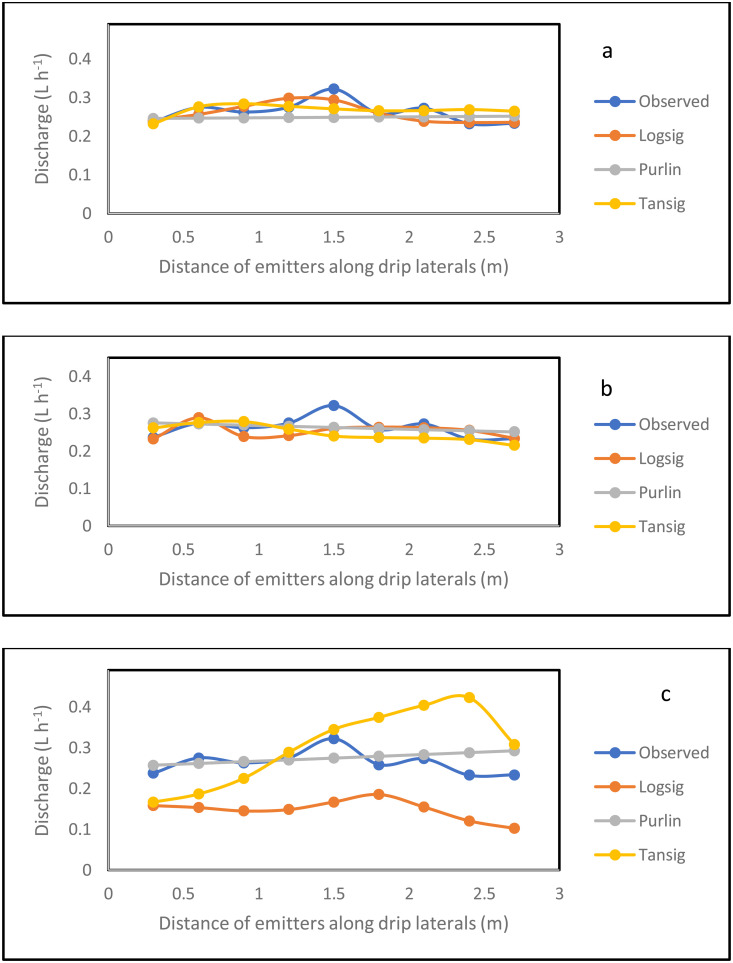
Simulation result for the emitter discharge along the drip laterals at 0.5 m elevation height and pipe length of 10 m (a), 11 m (b) and 13.5 m (c) pipe length using ANN.

**Fig 8:**
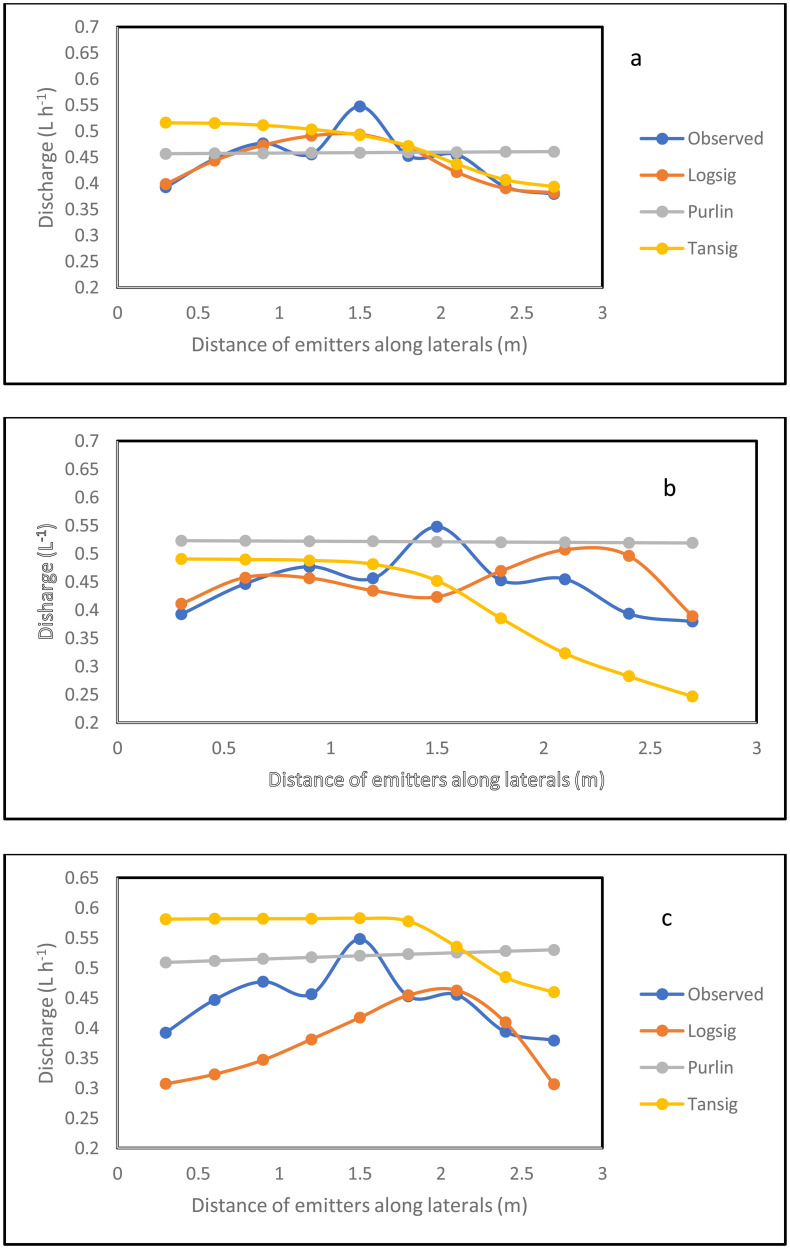
Simulation result for the emitter discharge along the drip laterals at 1 m elevation height and pipe length of 10 m (a), 11 m (b) and 13.5 m (c) pipe length using ANN.

The simulation result for the emitter discharge along the drip laterals at elevation heads of 0.1, 0.5 and 1 m are presented in Figs 6–8. For the simulation result from the ANN model to be well established, the simulation was tested at 3 different pipe lengths (10, 11.5 and 13 m) away from the fertigation source (Figs 6–8). The graphical illustration below showed that the Logsig and Tansig transfer functions mimic the field measured emitter discharge, with the purlin showing a kind of unchanged pattern (constant) value along the drip laterals. This observation was evident in all the elevation heads and pipe lengths. In addition, in most cases, the simulation gave a close result with the field obtained discharge measurement. The graphical illustration using the ANN also showed that the emitter discharge increases as the elevation head increases. This outcome showed that the ANN model could be used to simulate emitter discharge along drip laterals. Therefore, based on the closeness of the simulated result of the emitter discharge with the field measured values, there is a need to evaluate the performance of the ANN models using the different transfer functions (ANN Logsig, Tansig and Purlin).

The model evaluation results for the drip emitter discharge simulation are illustrated in Tables 5–7. The Tables show the model evaluation statistics in each of the elevation heads. The model simulated the emitter discharge along the drip emitter well. The performance of the trained model, in simulating the emitter discharge was evaluated with the statistical parameters (Tables 5–7). Considering all the transfer functions, the coefficient of determination ranged between 0.88 and 0.97 at the 10 m pipe length, 0.87–0.95 for the 11.5 m pipe length and 0.73–0.85 for the 13 m pipe length. This result showed that the model was well trained for the simulation of emitter discharge along the drip laterals. The high R^2^ value reported during training resulted to the precise simulation of the emitter discharge along the drip laterals during model validation and testing at the different pipe lengths of 10, 11.5 and 13 m. This high precision is confirmed by R^2^ value ranging between 0.61 and 0.90 during validation and between 0.64 and 0.89, while it ranged between 0.86 and 0.98 for all prediction. However, the lowest precision value of R^2^ in the range of 0.73–0.85 reported for the drip fertigation system could be attributed to pressure energy drop at the longest length (13 m). This drop in the pressure energy due to possible increased pipe length have resulted to variations in the emitter discharge along the drip laterals [91]. Thus, this variation in discharge measurement due to pressure energy drop is revealed in the reduced R^2^ value. Despite this, the R^2^ value of 0.73–0.85 produced by the ann at the 13 m pipe length could still be ranked good prediction. This is because about 73–85% variation in the emitter discharge is explained by the model.

**Table 5:** Simulation result for the emitter discharge at pipe length of 10 m.

	Statistics	Training	Validation	Testing	All
Logsig	R^2^	0.97	0.71	0.74	0.98
	MAE (L h^-1^)	0.0115	0.00104	0.00035	0.00649
	RMSE (L h^-1^)	0.0146	0.0322	0.018	0.013
	NRMSE	0.0741	0.0668	0.045	0.048
Purlin	R^2^	0.88	0.83	0.85	0.93
	MAE (L h^-1^)	0.0278	0.0021	0.0028	0.0142
	RMSE (L h^-1^)	0.0324	0.0457	0.0529	0.0263
	NRMSE	0.164	0.0949	0.126	0.0967
Tansig	R^2^	0.91	0.75	0.81	0.95
	MAE (L h^-1^)	0.0213	0.00281	0.000268	0.012
	RMSE (L h^-1^)	0.035	0.053	0.0164	0.0251
	NRMSE	0.179	0.11	0.039	0.0921

**Table 6:** Simulation result for the emitter discharge at pipe length of 11.5 m.

	Statistics	Training	Validation	Testing	All
Logsig	R^2^	0.95	0.61	0.68	0.93
	MAE (L h^-1^)	0.014	0.00411	0.0034	0.0109
	RMSE (L h^-1^)	0.0198	0.0641	0.0583	0.0256
	NRMSE	0.101	0.133	0.139	0.0942
Purlin	R^2^	0.9	0.64	0.85	0.94
	MAE (L h^-1^)	0.0198	0.0032	0.0111	0.0173
	RMSE (L h^-1^)	0.0355	0.0566	0.105	0.0377
	NRMSE	0.181	0.117	0.25	0.139
Tansig	R^2^	0.87	0.88	0.79	0.85
	MAE (L h^-1^)	0.0233	0.00294	0.013	0.0186
	RMSE (L h^-1^)	0.034	0.0542	0.114	0.0388
	NRMSE	0.173	0.113	0.271	0.142

**Table 7:** Simulation result for the emitter discharge at pipe length of 13 m.

	Statistics	Training	Validation	Testing	All
Logsig	R^2^	0.78	0.76	0.79	0.86
	MAE (L h^-1^)	0.0686	0.0138	0.00141	0.0327
	RMSE (L h^-1^)	0.0846	0.117	0.0376	0.0854
	NRMSE	0.23	0.243	0.09	0.151
Purlin	R^2^	0.89	0.63	0.85	0.93
	MAE (L h^-1^)	0.0303	0.00254	0.0126	0.021
	RMSE (L h^-1^)	0.0398	0.0505	0.112	0.058
	NRMSE	0.20	0.105	0.268	0.131
Tansig	R^2^	0.73	0.9	0.87	0.89
	MAE (L h^-1^)	0.0563	0.01157	0.00916	0.0209
	RMSE (L h^-1^)	0.0822	0.107	0.0957	0.058
	NRMSE	0.22	0.223	0.228	0.142

For the accuracy terms (MAE, RMSE and NRMSE), the result showed that the NRMSE ranged between 0.039 and 0.18 at 10 m pipe length. This showed that the prediction ranged between good and excellent at the 10 m pipe length. At the 11.5m pipe length, the NRMSE ranged between 0.094–0.27. This range of NRMSE indicated that it ranged from fair to excellent prediction, and this pattern was similarly observed at the 13 m pipe length.

Overall, in most cases, the lowest NRMSE was mostly observed using the Logsig transfer function. Similarly, the Logsig transfer function mostly produced the lowest MAE and RMSE. This outcome indicated that the Logsig transfer function is the best for the emitter discharge simulation, in term of accuracy. The tendency of the Logsig transfer function to outperform the Purlin and Tansig functions can be attributed to its ability to handle a wider range of input values and its non-linear nature which enables learning of complex patterns [104]. In similar application, Logsig transer functions outperformed Purlin and Tansig transfer functions in predicting the hydraulic conductivity of water in soils [59] and prediction of crop evapotranspiration [58,105]. For the precision, there were variations in the R^2^ values with all the transfer functions giving a good result. In similar areas of application, including hydrology, the ANN have been reported to accurately and precisely predict output from an instrument and system well [71,72].

Therefore, based on the high accuracy and precision level of the ANN, the model could be incorporated into the design of irrigation and fertigation by water resources engineer and practitioners to guide farmers on the effective management of irrigation and fertilizer. This is possible since the ANN predicted the amount of fertigation discharged at different elevation head, which can be adopted as one of the irrigation scheduling strategy means. This is important since an effective irrigation scheduling strategy could contribute to the maximization of crop yield and water use efficiency [106].

**Fig 9:**
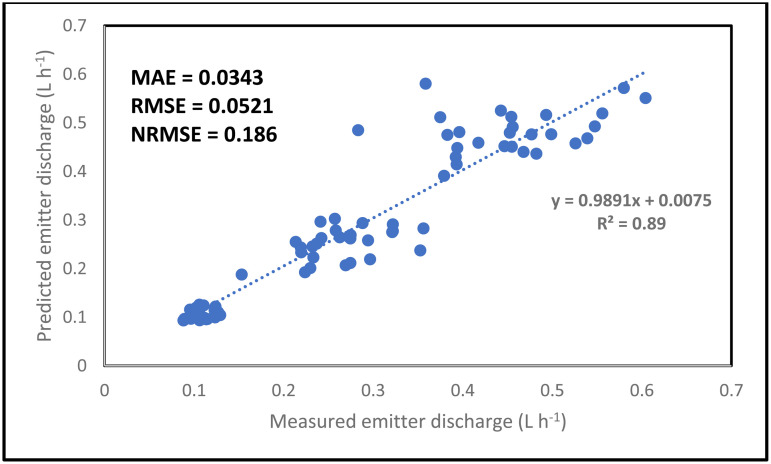
Overall simulation of emitter discharge along the drip laterals using the Logsig transfer function.

**Fig 10:**
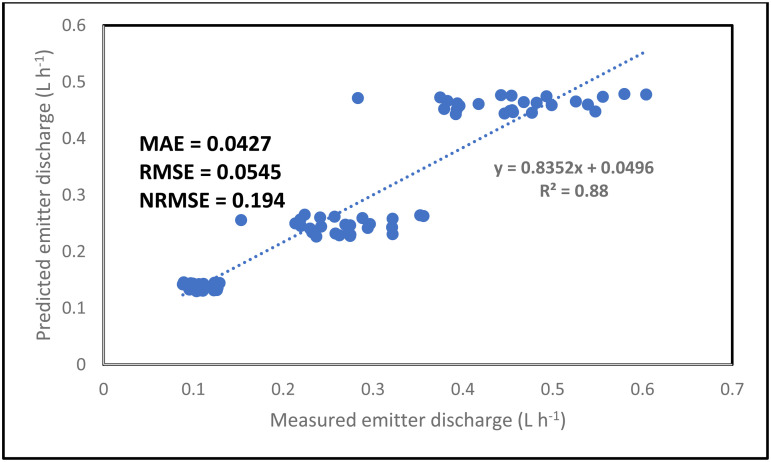
Overall simulation of emitter discharge along the drip laterals using the Purlin transfer function.

**Fig 11:**
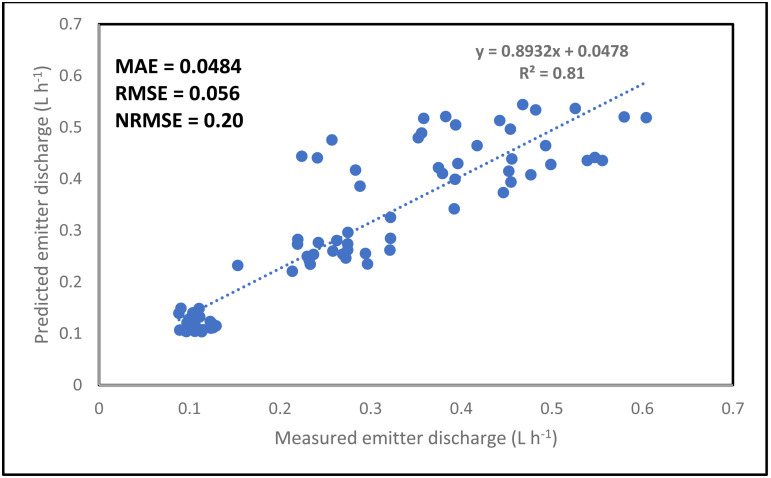
Overall simulation of emitter discharge along the drip laterals using the Tansig transfer function.

Considering the combination of all factors (pipe length, elevation heads and the distance of emitter points along the drip laterals) for the simulation of the drip emitter result, the ANN model predicted the emitter discharge accurately and precisely (Fig 9–11). The simulation was good since the NRMSE was between 10 and 20%. The NRMSE values indicated that the Logsig transfer function gave the best simulation in terms of accuracy. This can be confirmed as a result of the lowest RMSE, MAE and NRMSE values recorded for the Logsig transfer function. In addition, the ANN model precisely simulated the emitter discharge with the R^2^ value ranging between 0.81 and 0.89. Among all the transfer functions, the Logsig gave the best result in term of accuracy. Despite the varying input parameters (pipe length, distance of emitter points along the laterals and elevation head), the ANN still simulate the emitter discharge accurately. The Ability of the ANN model to accurately and precisely predict the emitter discharge along the drip laterals confirmed and justified the robustness and high capability of the ANN in simulating the emitter discharge along the drip laterals. The high magnitude of robustness and capability have been reported to be an added advantage in the simulation of output from different system and scenario [59,72]. This high-level predictability and robustness might have also supported the accurate and precise prediction of the emitter discharge along a drip lateral in this study.

## Conclusion

In this study, the lowest elevation head (0.1 m) was found to give the best hydraulic performance, with lower discharge. The rate of emitter discharge increased with increase in elevation head, while the discharge decreased with increase in the pipe length, although insignificantly (P > 0.05). Higher CU, AE and EU were observed at the lowest elevation head and at the longest pipe length due to gentle flow/discharge of water. There were interactive effects between the pipe length and elevation head on the emitter discharge and the CU, thus revealing the importance of investigating the combined effects of pipe length and elevation head on the hydraulic performance of a drip irrigation and fertigation system. This combination of factors has been scarcely reported in previous study, thus revealing the innovation in the study.

The development of ANN model for the simulation of emitter discharge along the drip laterals was accurate and precise, with the overall prediction producing NRMSE within 10 and 20%, while the R^2^ value was mostly above 0.8. The magnitude of the ANN prediction in term of prediction was reduced at the longer pipe length. This observation was attributed to pressure energy drop at this longer length. Despite this, the ANN showed its robustness in producing a good result at the longer length with R^2^ value ranging between 0.73 and 0.85. The ANN model also mimics the relationship between the elevation heads and the emitter discharge well, thus revealing an increase in discharge as the elevation head increases. Based on the good performance, ANN model should be incorporated into the design of drip irrigation system for effective management of irrigation water by the water resources engineer, to help farmers improve their productivity.

Among the transfer functions (Logsig, Purlin and Tansig) used for the prediction, the Logsig was the best in terms of accuracy and precision. Therefore, for further study, the Logsig transfer function is recommended for the prediction of emitter discharge along drip laterals. Moreso, the applicability of the ANN in conditions with longer pipe length and drip laterals length should be tested for further study. This would enable the determination and acceptability of the model for effective management of agricultural production in large scale. For the expansion of the present study, other models like support vector machine (SVM) and Adaptive Neuro-Fuzzy Inference System (ANFIS) should be incorporated, in predicting emitter discharge under drip-fertigation system.
